# The genus *Syntozyga* Lower (Lepidoptera, Tortricidae) in China, with descriptions of two new species

**DOI:** 10.3897/zookeys.1028.60297

**Published:** 2021-04-06

**Authors:** Wenxu Yang, Ruiqin Dong, Xueling Song, Haili Yu

**Affiliations:** 1 Shaanxi Key Laboratory for Animal Conservation, Northwest University, Xi’an, Shaanxi Province, 710069, China Northwest University Xi'an China; 2 Key Laboratory of Resource Biology and Biotechnology in Western China (Northwest University), Ministry of Education, Xi’an, Shaanxi Province, 710069, China Northwest University Xi'an China

**Keywords:** Barcodes, new species, Olethreutinae

## Abstract

Species of the genus *Syntozyga* Lower, 1901 (Lepidoptera, Tortricidae, Olethreutinae) from China are studied. *Syntozyga apicispinata***sp. nov.** and *S. similispirographa***sp. nov.** are described, *S. pedias* (Meyrick, 1920) is recorded for the first time from China, and *S. spirographa* (Diakonoff, 1968) is newly recorded from the Chinese mainland. Adults and genitalia are illustrated, and a distribution map of the Chinese species is given. Keys to identify the Chinese species of *Syntozyga* are provided. Species of the genus are well clustered in a neighbor-joining tree based on the sequence data of the COI gene. COI sequences corresponding to the new species and *S. spirographa* (Diakonoff, 1968) are submitted to BOLD.

## Introduction

The genus *Syntozyga*, a member of the subfamily Olethreutinae, was established by Lower (1901) with *S. psammetalla* Lower, 1901 as the type species. Diakonoff (1973) redescribed and figured *Syntozyga* species from south Asia, synonymized *Eleuthodema* Bradley, 1957 with *Syntozyga*, and placed *Syntozyga* together with five other related genera (*Bactra* Stephens, 1834, *Parabactra* Meyrick, 1910, *Henioloba* Diakonoff, 1973, *Bubonoxena* Diakonoff, 1968, and *Cyclacanthina* Diakonoff, 1973) in Bactrae, one of his newly proposed 12 subtribes of the tribe Olethreutini. The taxonomic status of this genus group has been in dispute (Kuznetsov and Stekolnikov 1977; Razowski 1989; Dang 1990; Horak and Brown 1991; Horak 2006; Sofonkin 2007; Gilligan et al. 2018). Horak (2006) synonymized *Bubonoxena* and *Cyclacanthina* with *Syntozyga*, provided extensive descriptions, references, and pictures of *Syntozyga* adults, and discussed its relationships and taxonomy. Up to now, *Syntozyga* has contained 19 species with representatives in the Oriental, African, and Australian faunas (Lower 1901; Meyrick 1907, 1911, 1920, 1921; Turner 1916, 1945, 1946; Diakonoff 1968, 1971, 1973; Kuznetsov 1988a, b; Horak 2006; Aarvik 2008; Razowski and Trematerra 2010; Razowski and Wojtusiak 2012; Razowski 2014; Gilligan et al. 2018 ; Razowski and Bassi 2018). The ecology and host plants of *Syntozyga* are poorly known. Fletcher (1932) reported from India that the larvae of *S. ephippias* (Meyrick, 1907) are borers in stems of *Commelina benghalensis* (Commelinaceae). *Syntozyga sedifera* (Meyrick, 1911) was reared from *Glochidion* sp. (Phyllanthaceae) in Australia (Brown et al. 2008).

Seven full-length barcodes (COI gene, 658 bp) representing three species of *Syntozyga* are available through GenBank (accessed 20 October 2020); these are given in Suppl. material 1: Table S1.

Up to now, no species of *Syntozyga* has been known from the Chinese Mainland, but *Syntozyga spirographa* (Diakonoff, 1968) has been recorded from Taiwan (Heppner and Inoue 1992). The purpose of this paper is to provide faunistic data on the *Syntozyga* species in China. Herein, we add three species of *Syntozyga* to the reported Chinese fauna, including two new species, *S. apicispinata* sp. nov. from Tibet and Yunnan and *S. similispirographa* sp. nov. from Yunnan, and one species newly recorded for China, *S. pedias* (Meyrick, 1920) from Hainan Island, in addition to numerous records for *S. spirographa* from the Chinese mainland. Eleven COI sequences (658 bp) representing three species (*S. apicispinata* sp. nov., *S. similispirographa* sp. nov., and *S. spirographa*) were obtained (Suppl. material 1: Table S1).

## Material and methods

The material examined in this study was collected by using light traps. Terminology for forewing pattern follows Brown and Powell (1991), as refined by Baixeras (2002). Methods for genitalia dissection follow Li (2002). The abdomen and genitalia were slide-mounted using Canada balsam. The specimens were examined with an Olympus SZX71 stereomicroscope. All images were captured with a digital microscope (VHX-5000).

Total genomic DNA was extracted with the Genomic DNA Extraction Kit (Tiangen Biotech., Beijing, China) from legs removed from dried adult specimens according to the manufacturer’s instructions. Genomic DNA was eluted into 50 μL TE buffer and stored in a freezer (−20 °C). The target fragments of COI were amplified as described by Hebert et al. (2004). The sequences were aligned using ClustalW in MEGA 7 (Kumar et al. 2016) and the genetic distance was analyzed using the Kimura 2-Parameter model. The sequence data obtained in this study have been deposited in GenBank (Accession numbers: MW187146–MW1871556) and in the BOLD database (Ratnasingham and Hebert 2007) (Voucher numbers: TORTR001-20–TORTR011-20).

The types of the new species are deposited in the Insect Collection of the Northwest University, Xi’an, China (NWU).

## Results

### Syntozyga

Taxon classificationAnimaliaLepidopteraTortricidae

Lower, 1901

ACDE2BDF-391E-5CC1-85D1-29D8D302B6DD

Syntozyga Lower, 1901: 70. Type species: Syntozyga psammetalla Lower, 1901.
Eleuthodema Bradley, 1957: 95. Type species: Polychrosis pedias Meyrick, 1920.
Bubonoxena Diakonoff, [1968]: 40. Type species: Bubonoxena spirographa Diakonoff, [1968].
Cyclacanthina Diakonoff, 1973: 337. Type species: Cyclacanthina episema Diakonoff, 1973.

#### Remarks.

Superficially, adults of *Syntozyga* are similar to many species of Olethreutini, especially members of the subtribe Olethreutae, but they lack secondary sexual structures on the wing and legs in the male, that makes it easy to identify them from Olethreutae. In the male genitalia, *Syntozyga* is readily separated from most genera of Olethreutini by the absence of an uncus and by strongly reduced socii. In the Olethreutinae, reduction of the appendages of the tegumen is also found in the tribe Grapholitini, but their valvae are extremely simple in structure and evenly spined. In contrast, *Syntozyga* is characterized by a valva with a broad sacculus projecting distally and carrying varied vestiture such as spine clusters and strong bristles or thorns; sometimes a naked lobe (or two) is present between the cucullus and the apex of sacculus (*S. cerchnograpta* Razowski, 2014, *S. triangulana* Aarvik, 2008, *S. spirographa*, *S. similispirographa* sp. nov., and *S. apicispinata* sp. nov.). Detailed descriptions of the morphology of *Syntozyga* were provided by Diakonoff (1968, 1973) and Horak (2006).

### Key to Chinese species of *Syntozyga* based on habitus

**Table:** 

1	Forewing with apex rounded, markings dark blackish brown, sparsely dusted with pale yellow, media fascia not interrupted medially	***S. pedias***
–	Forewing with apex projecting, or nearly square, markings dark brown, densely suffused with pale yellow, media fascia interrupted medially	**2**
2	Forewing with indistinct silvery striae between markings on distal part; hindwing brown	***S. apicispinata* sp. nov.**
–	Forewing with distinct leaden striae between markings on distal part; hindwing grayish brown	**3**
3	Hindwing with termen nearly straight, not concave	***S. similispirographa* sp. nov.**
–	Hindwing with termen concave at M_2_	***S. spirographa***

### Key to Chinese species of *Syntozyga* based on male genitalia

**Table:** 

1	Valva without spine clusters or strong thorns from ventral edge, distal part broadly rounded, broader than basal half	***S. apicispinata* sp. nov.**
–	Valva with a cluster of long spines or a strong thorn from ventral edge, distal part narrower than basal half	**2**
2	Valva bearing a strong thorn on apical margin of sacculus with any associated spines shorter than thorn	***S. pedias***
–	Valva with a cluster of long spines on apical margin of sacculus	**3**
3	Valva with median part more than 2 times apical part in width; basal excavation short, distal edge reaching middle of sacculus; a naked triangular lobe adjacent to the spine patch of cucullus ventrally, with its base distant from the cluster of long spines on sacculus	***S. similispirographa* sp. nov.**
–	Valva with median part less than 1.5 times apical part in width; basal excavation broad, distal edge reaching 2/3 of sacculus; a naked triangular lobe between the spine patch of cucullus and the cluster of long spines on sacculus, its base adjacent to the spine cluster	***S. spirographa***

### Syntozyga pedias

Taxon classificationAnimaliaLepidopteraTortricidae

(Meyrick, 1920)

C80E5411-EC82-567A-B857-D81EA1ABAFA7

(Figs 1 , 5 , 9)

Polychrosis pedias Meyrick, 1920: 347 (♀, Bengal).
Eucosma familiaris Meyrick, 1921: 153 (♂♀, Java).
Lobesia pedias : Clarke, 1958: 472 (fig. 2b of holotype).
Syntozyga pedias Diakonoff, 1973: 341 (Cheng [sic] Mai, Kuala Lumpur, Borneo, Bali, Celebes, Western New Guinea) (figs 515, 531–533, 537, 546).

#### Material examined.

**China, Hainan Prov.**: 1♂, Datian Reserve, 19°06'N, 108°47'E, alt. 25 m, 1 Jul. 2009, leg. Zhaohui Du and Linlin Yang.

#### Distribution.

India, Indonesia, Thailand, Malaysia, China (Hainan).

#### Note.

This species is newly recorded for China. The male genitalia accord well with those of the lectotype of *Eucosma familiaris* from Java figured by Diakonoff (1973).

### Syntozyga apicispinata

Taxon classificationAnimaliaLepidopteraTortricidae

Yang & Yu sp. nov.

7B9421E6-3076-5276-AB57-24CD0F76E380

http://zoobank.org/31C77B40-5FA3-4633-B34C-3E3C6E01EB87

(Figs 2 , 6 , 10b)

#### Type material.

***Holotype*: China, Tibet**: Motuo County, Bengbeixiang, 29°24'N, 95°17'E, alt. 990 m, 12 Aug. 2017, leg. Mujie Qi and Xiaofei Yang, genitalia slide no. YWX18246. ***Paratypes*: China, Tibet**: 6♂, same data as holotype; 2♂, same data as holotype except 29°25'N, 95°18'E, alt. 810 m, 15 Aug. 2017; 3♂; same data as holotype except alt. 750 m, 31 Jul.–1 Aug. 2018, leg. Mujie Qi; **Yunnan Prov.**: 1♂, Xishuangbanna, Guanping, 22°46'N, 100°59'E, alt. 1200 m, 18 Aug. 2005, leg. Yingdang Ren; 1♂, Xishuangbanna, Yexianggu, 22°10'N, 100°52'E, alt. 760 m, 11 Jul. 2015, leg. Kaijian Teng and Xia Bai; 1♂, Mengla, Bubeng, 21°36'N, 101°35'E, alt. 650 m, 31 Jul. 2020, leg. Yongyan Li and Wenxu Yang.

#### BOLD voucher no.

TORTR001-20, TORTR008-20, TORTR009-20, TORTR010-20, TORTR011-20.

#### Diagnosis.

Externally, *S. apicispinata* is similar to *S. spirographa* (Diakonoff, 1968) and *S. similispirographa*, but the relatively paler striae derived from the costal strigulae and the brown or pale brown hindwing can be used to separate it from the latter two species. The male genitalia of *S. apicispinata* are very different from those of *S. spirographa* and *S. similispirographa.* It can be best distinguished by a distally broad, ovate valva, a finger-like prominence on costa, the absence of a cluster of long spines on apex of sacculus, the presence of a cluster of short spines below apex of cucullus and a stout phallus with minute cornuti apically. Within the genus, this species resembles *S. episema* (Diakonoff, 1973) from Java and Sumba in the male genitalia, but it can be separated from *S. episema* by the valva with its ventral edge not concave in the middle and without long thorns, a sacculus with a sharp, triangular prominence on ventral margin of basal excavation, and a cucullus bearing a cluster of short spines below apex. In *S. episema*, the ventral edge of the valva is concave medially and is densely set with several long thorns, there is no prominence on the ventral margin of the basal excavation and the apical part of cucullus has no prominent spine clusters. *Syntozyga apicispinata* also resembles *S. negligens* (Diakonoff, 1973) from Java and Sulawesi in the male genitalia, but it can be distinguished from the latter species by the valva with its apical part broader than its basal part and having a sharp triangular prominence on the ventral margin of the basal excavation. In *S. negligens*, the apical part of the valva is narrower than its basal part, and there is no projection from the ventral margin of the basal excavation.

#### Description.

**Male** (Fig. 6). Forewing length 5.5–6.5 mm. ***Head*** (Fig. 2): ocellus well developed; chaetosemata present. Frons smooth, creamy white; vertex roughly scaled, creamy. Antenna with scape creamy, flagellum dark brown, dusted with pale brown distally. Labial palpus porrect, creamy suffused with pale brown, median segment expanded slightly, terminal segment 1/2 times of median segment in length, brown apically.

***Thorax*:** creamy, dusted with brown and dark brown medially; posterior crest distinct. Collar creamy. Tegula dark brown basally and creamy suffused pale brownish yellow distally. Hind tibia not expanded, without hair pencil in male. Forewing elongate subrectangular, costa evenly arched, apex blunt, termen oblique; upperside general ground color creamy, with markings dark brown and dusted with pale brownish yellow; costal strigulae creamy white, the fifth and sixth pairs suffused with gray brown; basal fascia broken into two parts, a small blotch on costa and an obliquely arched streak extending to inner margin of wing; subbasal fascia consisting of three parts, an oblique blotch on costa, an irregular blotch between upper edge of cell and 1A+2A, and an indistinct spot or shadow on base of dorsum; a discontinuous pale streak present between pairs of strigulae three and four, extending to dorsum; media fascia broken medially, anterior half fused with costal part of postmedian fascia forming a conspicuous subtriangular patch on costa, extending to midwing, lower part present between midwing and distal half of dorsum, indistinct and intricate, confluent with the surrounding spots and pretornal patch; postmedian fascia obliquely arched, indistinct between 2/3 length of R_2_ and midlength of R_5_, lower part elongate, extending to termen between M_2_ and CuA_1_; a short line present on costa between strigulae seven and eight; preterminal fascia and terminal fascia as two elongate streaks confluent to termen; cilia pale brown fixed with dark brown and creamy; underside yellowish brown to brown. Hindwing subtriangular, brown; cilia pale brown; underside pale brown.

**Figures 1–4.**
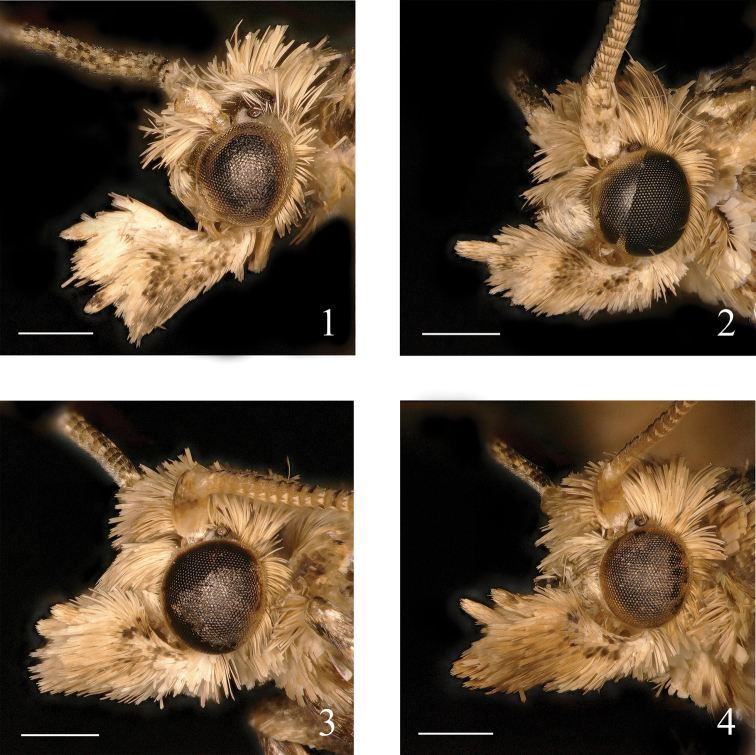
Heads of *Syntozyga***1***S. pedias*, Hainan (male) **2***S. apicispinata* sp. nov., Tibet (male holotype) **3***S. spirographa*, Hainan (male) **4***S. similispirographa* sp. nov., Yunnan (male holotype). Scale bars: 0.5 mm.

***Abdomen*:** male genitalia (Fig. 10): tegumen low, with ear-like shoulders laterally and a moderately broad, rounded triangular top with some hairs; vinculum a sclerotized band, strong, somewhat W-shaped; uncus absent; socii strongly vestigial, indicated by a narrow patch of sparse spines on ventrodistal margin of tegumen; gnathos a weakly sclerotized band; valva broad, oval, costa with a folded ridge from the base to the apex, a short finger-like prominence protruding from the middle of the ridge bearing long thorns basally and apically, basal excavation large, sacculus narrow with three sclerotized, naked lobes (Fig. 10a): one small subtriangular sitting on the ventral margin of the basal excavation medially, the other two large, rounded, occupying the distal half of the sacculus, positioned right next to each other, the inner one with its edge smooth, the outer one with its edge serrated; a patch of thin, short bristles present beyond the basal excavation; cucullus membranous, roughly as long as basal part of valva, broadly rounded, with bristles along margin, a short conspicuous lobe proximal to apical margin, carrying a dense cluster of spines; phallus short and stout, with tiny spiny cornuti apically (Fig. 10b).

**Female** unknown.

#### Etymology.

The specific name is derived from the Latin prefix *apici*- (= apical) and the adjective *spinatus* (= spiny), indicating the apical spine cluster of the valva.

### Syntozyga spirographa

Taxon classificationAnimaliaLepidopteraTortricidae

(Diakonoff, 1968)

A4326680-2694-5DC5-8CAA-D8E6D5216D10

(Figs 3 , 7a , 11 , 13)

Bubonoxena spirographa Diakonoff, 1968: 66 (♂, Luzon) (figs 82, 103–104, 540).
Syntozyga spirographa : Diakonoff, 1973: 346 (North Celebes, West Sumatra) (figs 516, 547).

#### Material examined.

**China, Chongqing**: 2♂, Mt. Jinfo, 29°02'N, 107°11'E, alt. 1100 m, 5 May 2013, leg. Xiaofei Yang; **Fujian Prov.**: 1♀, Xiamen, 24°35'N, 117°55'E, alt. 40 m, 11 Jul. 2010, leg. Bingbing Hu and Jing Zhang; 1♀, same data except 28 Jun. 2012, leg. Zhibo Wang; **Guangxi Prov.**: 4♂, Yizhou, Pingxin village, 24°40'N, 108°21'E, alt. 150 m, 16 Aug. 2011, leg. Shulian Hao and Yinghui Sun; 8♂, 1♀, Pingxiang, 22°01'N, 106°51'E, alt. 190 m, 300 m, 550 m, 26 Jun.–2 Aug. 2011, leg. Bingbing Hu; 1♂, same data except alt. 280 m, 15 May 2012, leg. Xiaofei Yang; 1♂, same data except 23 Mar. 2013; **Guizhou Prov.**: 1♂, Libo, Banzhai village, 25°13'N, 108°10'E, alt. 530 m, 9 Aug. 2018, leg. Meiling Zheng, Jiaqi Deng and Xiaoju Zhu; 1♂, same data except alt. 510 m, 24 Jul 2019, leg. Mengran Xing, Baixue Zhao and Hao Sun; **Hainan Prov.**: 1♂, Lingao County, Mt. Duowenling, 19°47'N, 109°45'E, alt. 120 m, 9 May 2009, leg. Bingbing Hu and Qing Jin; 2♂, Lingshui, Mt. Diaoluo, 18°39'N, 109°56'E, alt. 80 m, 21–22 Apr. 2013, leg. Yinghui Sun, Wei Guan and Tengteng Liu; 1♂, Changjiang County, Qichazhen, 19°6'N, 109°4'E, alt. 130 m, 4 May 2013, leg. Yinghui Sun, Wei Guan and Tengteng Liu; 1♂, Baisha County, Hongxin village, 19°40'N, 109°31'E, alt. 460 m, 1 Jul. 2014, leg. Peixin Cong, Linjie Liu and Sha Hu; 1♂, Ledong, Jianfengzhen, 18°41'N, 108°46'E, alt. 50 m, 12 Jul. 2014, leg. Peixin Cong, Linjie Liu and Sha Hu; 1♂, Dongfang, Datian village, 19°3'N, 108°50'E, alt. 120 m, 2 Jan. 2018, leg. Mujie Qi and Shuai Yu; 1♂, Dongfang, Lemei village, 19°8'N, 108°84'E, alt. 80 m, 3 Jan. 2018, leg. Mujie Qi and Shuai Yu; **Yunnan Prov.**: 3♂, 1♀, Mengla, River Nanla, 21°59'N, 101°58'E, alt. 650 m, 12–14 Jul. 2013, leg. Shurong Liu, Yuqi Wang and Kaijian Teng.

#### BOLD voucher no.

TORTR003-20, TORTR004-20, TORTR005-20, TORTR006-20.

#### Distribution.

Philippines, Indonesia, China (Chongqing, Fujian, Guangxi, Guizhou, Hainan, Yunnan, Taiwan).

#### Notes.

In China, previously Taiwan was the only area where *S. spirographa* had been collected (Heppner and Inoue 1992). In the present study, we identified this species from six provinces of the Chinese mainland for the first time, with a distribution to the south of latitude 30°N and between longitudes 100°E and 118°E.

### Syntozyga similispirographa

Taxon classificationAnimaliaLepidopteraTortricidae

Yang & Yu sp. nov.

1C0A7163-E5E0-5FB3-9BFE-B43F0369BC94

http://zoobank.org/DC2336BD-4545-47A5-ADE7-E0A24270AEE3

(Figs 4 , 8a , 12a , 14)

#### Type material.

***Holotype*: China, Yunnan Prov.**: 1♂, Longling, Xiaoheishan Reserve, 24°52'N, 98°84'E, alt. 1970 m, 30 Jul. 2015, leg. Kaijian Teng and Baixia, genitalia slide no. YWX18337. ***Paratypes*: China, Yunnan Prov.**: 3♂, same data as holotype except 17–19 Jul. 2013, leg. Shurong Liu, Yuqi Wang and Kaijian Teng; 1♂, Pu’er, Mt. Yunpan, 22°41'N, 100°39'E, alt. 1400 m, 9 Jul. 2013, leg. Zhenguo Zhang; 1♂, same data except alt. 1620 m, 6 Aug. 2020, leg. Wenxu Yang; 1♂, Pu’er, Sun River Reserve, 22°35'N, 101°06'E, alt. 1600 m, 11 Jul. 2013, leg. Zhenguo Zhang; 16♂, same data except alt. 1450 m, 13 May–3 Jun. 2014; 8♂, 2♀, Pu’er, Sun River Reserve, 22°60'N, 101°11'E, alt. 1630 m, 6–7 Jul. 2013, leg. Shurong Liu, Yuqi Wang and Kaijian Teng; 1♂, Pu’er, Sun River Reserve, 22°68'N, 101°03'E, alt. 1450 m, 6 Jul. 2015, leg. Kaijian Teng; 1♀, Jingdong, 22°60'N, 101°11'E, 18 Aug. 2009, leg. Xicui Du.

#### BOLD voucher no.

TORTR002-20, TORTR007-20.

#### Diagnosis.

*Syntozyga similispirographa* is very similar to *S. spirographa* in external appearance. The shape of the hindwing termen is the only stable superficial character to identify the two species, it is concave at M_2_ in *S. similispirographa* and straight in *S. spirographa.* However, the genitalia in both sexes are distinct. In *S. similispirographa*, the apical part of the cucullus is obviously narrow, less than half the width of the median part of the valva; the basal excavation is short, reaching only to mid length of sacculus; the naked triangular lobe is adjacent to the spine patch of cucullus, its base is distant from the cluster of long spines of sacculus; the phallus is moderately wide, with its width at middle about 1/5 its length. In *S. spirographa*, the apical part of cucullus is rounded, more than 2/3 the width of the median part of the valva; the long basal excavation is extending to 2/3 length of sacculus; the naked, triangular lobe is situated between the spine patch of cucullus and the cluster of long spines on sacculus, its base is adjacent to the spine cluster; the phallus is slender, its width in middle is about 1/8 its length. In the female genitalia of *S. similispirographa* the sterigma is complex and not flat, including the anteriorly raised, spinulose lamella antevaginalis and two naked, concave lateral extensions. In *S. spirographa*, the sterigma is relatively uniform structure, indicated by the large, wholly spinulose lamella antevaginalis which is extending posteriorly and laterally. Externally *S. similispirographa* also resembles *S. apicispinata*, but the leaden striae on the forewing and the relatively darker, more grayish hindwing separate it from the latter species.

**Figures 5–8.**
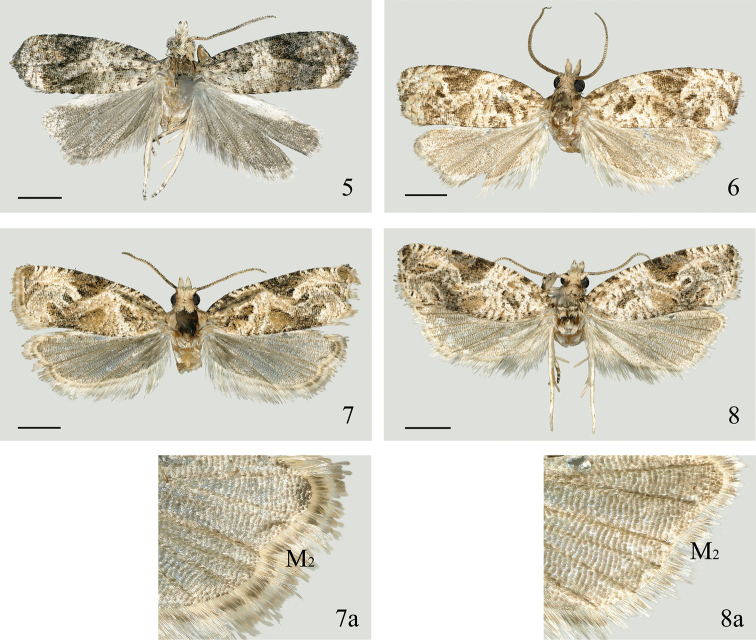
Adults of *Syntozyga***5***S. pedias*, Hainan (male) **6***S. apicispinata* sp. nov., Tibet (male holotype) **7***S. spirographa*, Hainan (male) **7a** termen of hindwing **8***S. similispirographa* sp. nov., Yunnan (male holotype) **8a** termen of hindwing. Scale bars: 1.5 mm.

#### Description.

**Adult** (Fig. 8). Forewing length 5.0–7.0 mm in male, 5.5–7.0 mm in female. ***Head*** (Fig. 4): ocellus well developed; chaetosemata present. Frons smooth, white; vertex roughly scaled, creamy. Antenna with scape creamy, flagellum brown. Labial palpus porrect to ascending, pale creamy, median segment widened in distal half, dusted with brown and dark brown laterally, terminal segment slender, about half length of median segment, sparsely or slightly dusted with brown.

***Thorax*:** creamy, dusted with brown medially; posterior crest distinct. Collar creamy. Tegula dark brown basally and creamy suffused with yellow and pale brown distally. Hind tibia not expanded, without hair pencil in male. Forewing elongate subrectangular, costa lightly curved throughout, apex rounded-rectangular, termen oblique; upperside ground color creamy white, markings dark brown dusted densely with yellow; costal strigulae creamy white, basal two pairs suffused with yellow; basal fascia broken, indicated by a small blotch below base of costa and a short streak on inner margin of wing; subbasal fascia represented by a darker brown spot on costa, an oblique blotch between 2/5 length of cell and 1/3 length of 1A+2A, and several faint ripples above the basal part of dorsum; leaden striae from strigulae three and four distinct on disc of cell and extending obliquely to 2/3 length of dorsum and confluent with significant leaden striae from strigulae five and six below distal part of lower edge of cell, thus separating media fascia; upper part of media fascia fused with costal part of postmedian fascia, forming a large, somewhat inverted triangular patch, lower part of media fascia between distal part of cell and dorsum, oblique triangular; a darker brown dot on outer edge of cell often present; pretornal patch distinguishable, oblique elongate; lower part of postmedian fascia oblique, present between midlength of R_4_ and 2/3 length of M_2_, surrounded by leaden striae from distal five pairs of costal strigulae, which broadly extend to tornus; preterminal fascia indistinguishable; terminal fascia a narrow streak, extending along termen to M_2_; cilia pale brown mixed dark brown and creamy; underside yellowish brown to brown. Hindwing subtriangular, termen nearly straight (Fig. 8a); pale gray-brown to pale brown; cilia gray to pale brown; underside pale gray to pale brown.

***Abdomen*:** male genitalia (Fig. 12): tegumen low, with a rounded or triangular top; vinculum a narrow band; socii indicated by a narrow spine patch, below margin of tegument apex; gnathos a weakly sclerotized band; valva somewhat triangular, basal half gradually widening, medially about twice as wide as distal part of cucullus; basal excavation short, distal edge reaching about 1/2 length of sacculus; sacculus broad, almost naked except for prominent and rounded apex bearing a cluster of very long spines; cucullus triangular, gradually narrowed to apex, apex rounded-acute, disc area with an elongate semicircular patch of spines, an acute triangular lobe proximate to this spine patch ventrally, naked, with its base distant from the cluster of long spine on sacculus (Fig. 12a); a rounded lobe projecting along basal half of ventral margin, short, sparsely set with fine spines; apex with dense spines dorsally; phallus moderately long and wide, tapering towards apex, without cornuti. Female genitalia (Fig. 14): papilla analis narrow and elongate. Sternum 7 weakly sclerotized and with a concave hind margin. Sterigma an irregularly shaped complex structure, lamella antevaginalis broad, spinulose, raised medially and with a longitudinal groove anteriorly, ostium flanked by two concave, naked and petal-shaped pockets. Ductus bursae membranous and smooth, with weakly sclerotized and ill-defined colliculum, faintly widening towards corpus bursae. Corpus bursae ovate, weakly granular, without signa.

**Figures 9–12.**
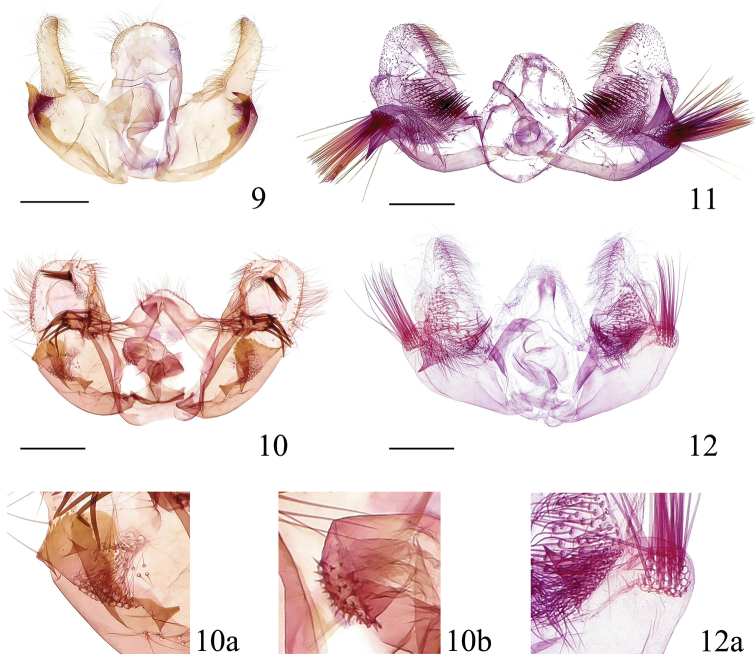
Male genitalia of *Syntozyga***9***S. pedias*, Hainan, slide no. YHL07156 **10***S. apicispinata* sp. nov. (holotype), Tibet, slide no. LYY18320 **10a** naked lobes **10b** cornuti **11***S. spirographa*, Hainan, slide no. LKL15037 **12***S. similispirographa* sp. nov. (paratype), Yunnan, slide no. YWX18337 **12a** naked lobe. Scale bars: 0.5 mm.

#### Etymology.

This specific name is derived from the Latin prefix *simil*- (= similar) and the taxon name *spirographa*, referring to the similarity of this new species to *S. spirographa* (Diakonoff).

**Figures 13, 14.**
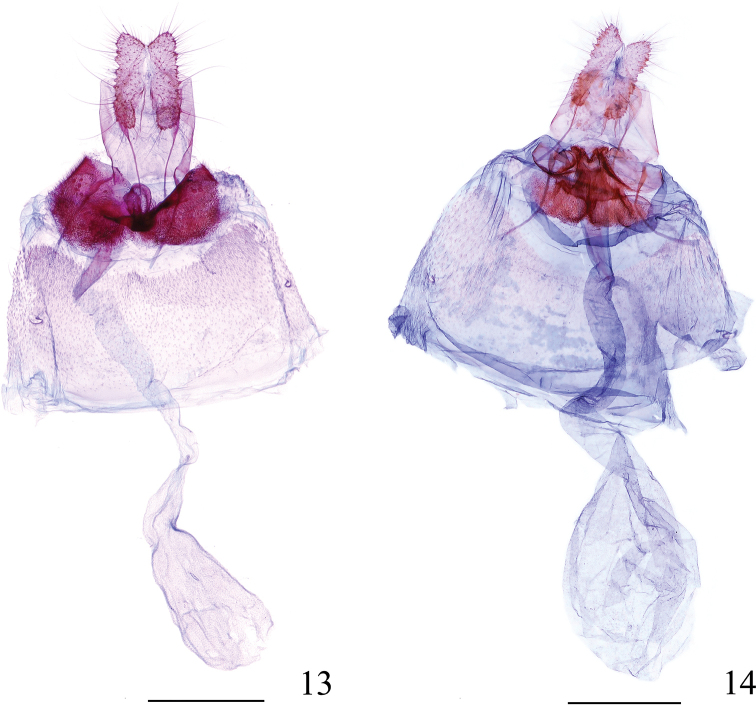
Female genitalia of *Syntozyga***13***S. spirographa*, Fujian, slide no. YWX18338 **14***S. similispirographa* sp. n. (paratype), Yunnan, slide no. YWX18342. Scale bars: 0.5 mm.

##### Molecular data analyses

**Figure 15.**
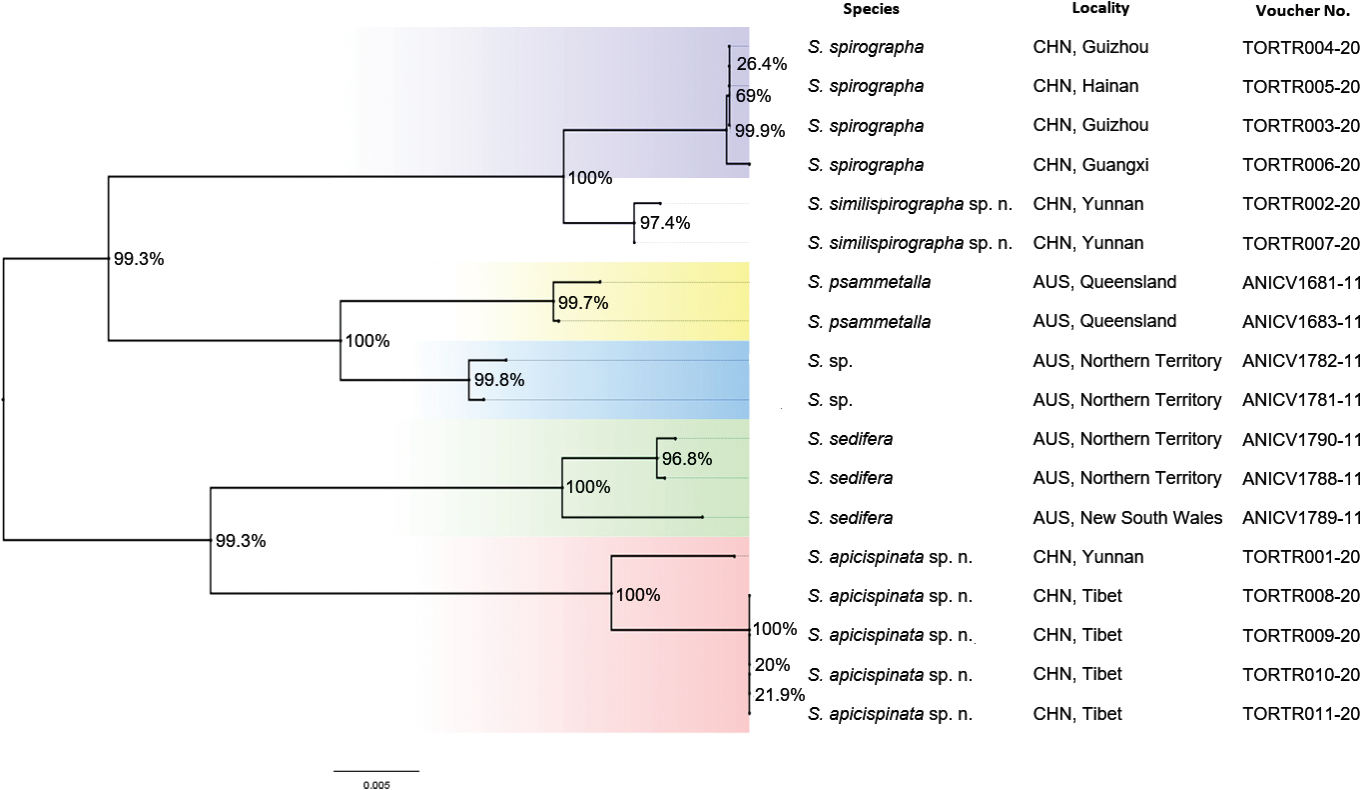
Neighbor-joining Tree, based on DNA barcodes of *Syntozyga* species. Numbers above/below branches refer to bootstrap proportions.

All results are based on 18 COI gene sequences belonging to the six species listed in Suppl. material 1: Table S1. Of these, 11 sequences were newly obtained, and seven sequences were taken from GenBank. All sequences are 658 bp in length, and the genetic distances are presented in Suppl. material 1: Table S2. The interspecific genetic distances within the six species varied from 1.4% (*S. spirographa* to *S. similispirographa*) to 8.9% (*S. apicispinata* to *S. spirographa*), and the average divergence is 6.5%. The intraspecific divergence in the six species (including the unidentified species, *Syntozyga* sp.) varied from 0 to 1.5% (*S. sedifera*, individual of *S. apicispinata* from Yunnan to those from Tibet) and the average divergence is 0.4%. A neighbor-joining (NJ) tree (Fig. 15) covering the above six species was generated for facilitating species delimitation. Six well-supported clades of *Syntozyga* sequences are revealed by the NJ tree, each clade of which represents one species.

**Figure 16.**
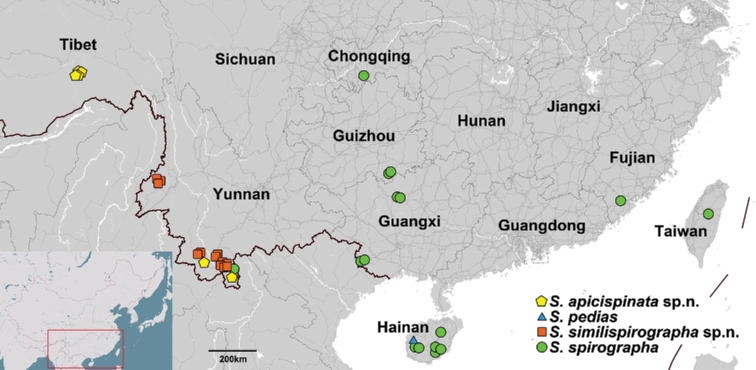
Distribution of *Syntozyga* species in China.

## Discussion

In Lepidoptera, barcode divergence of approximately 2% is often congruent with morphology-based species-level identifications (Hajibabaei et al. 2006; Zahiri et al. 2014). In this present study, we found that *S. similispirographa*, with a similar appearance to *S. spirographa*, shows a low divergence (1.4%) in their COI barcodes. However, distinct and consistent differences between genital structures in both sexes of the two species can be observed, such as shape and size of basal excavation, width of cucullus, location of the naked, triangular lobe near the cluster of long spines in the male, and sterigma shape in the female (Figs 11–14). Low interspecific genetic divergence of congeneric species pairs may mean recent origin or mitochondrial introgression (Muñoz et al. 2011; Zahiri et al. 2014), and it is risky to draw conclusions about species identification from mtDNA sequence alone (Ritter et al. 2013; Lukhtanov et al. 2015). Therefore, based on significant morphological difference between the two populations in China, in contrast to the consistent genitalia morphology of *S. spirographa* across its wide range, we described individuals from Sun River Reserve and Xiaoheishan Reserve, Yunnan Province, as a distinct species, *S. similispirographa* sp. nov.

Compared to other members of Olethreutini, the Chinese species of *Syntozyga* have a rather long and slender terminal segment of their labial palpus, which is clearly projected, especially in the two new species.

Three species, *S. spirographa*, *S. similispirographa*, and *S. apicispinata*, are distributed in an area 200 km across in southern Yunnan (Fig. 16). They share the forewing pattern characterized by complicated markings of sinuate pattern elements, with a large, dark-brown, inverted triangle over the central 1/4 of the costa. But while they are difficult to distinguish externally, they have two distinct configurations of the male genitalia. *Syntozyga apicispinata* is characterized by a well-sclerotized sacculus with strongly sclerotized edge, a wide, rounded cucullus with a spined process from its costa and a stout phallus, and has a close relationship with three species described by Diakonoff (1973) (*S. episema*, *S. negligens* and *S. monosema*) from Indonesia and Sri Lanka. In *S. spirographa* and *S. similispirographa*, the male has an expanded sacculus bearing a cluster of very long spines distally, a distally narrowing cucullus with a central spine patch basally and a slender phallus, similar to the south Asian species, *S. ephippias*, and the African *S. tryphera* Razowski & Wojtusiak, 2012 and *S. cerchnograpta*. Accordingly, there is high genetic divergences between *S. apicispinata* and the other two Chinese species, 8.3% to *S. spirographa* and 8.9% to *S. similispirographa.*

The distribution of the four species of *Syntozyga* in China is shown in Fig. 16. *Syntozyga spirographa* has a relatively broad distribution in southern China, but specimens from different localities agree in appearance and genital structure. There is a slight genetic difference (0.2%) between material from Yizhou (Guangxi Prov.) and the other two localities, Libo (Guizhou Prov.) and Dongfang (Hainan Prov.). This is somewhat puzzling as Yizhou is near Libo and located between Libo and Dongfang. Knowledge of host plant association might provide an answer, so further examination is desirable. High intraspecific divergence was observed in the discontinuously distributed *S. apicispinata* (1.5%), collected in southern Tibet and southern Yunnan, respectively, separated by 900–1000 km. However, no obvious genital variation was found between the two populations. *Syntozyga similispirographa* has a narrow distribution in two locations of southwest Yunnan, the Sun river and the Xiaoheishan reserves, separated by approximately 400–500 km. The genetic divergence between two male specimens collected in these two localities is 0.2%. Although *S. pedias* is distributed widely in southeast Asia (India, Thailand, Indonesia), it is only known from Hainan Island in China.

## Supplementary Material

XML Treatment for Syntozyga

XML Treatment for Syntozyga pedias

XML Treatment for Syntozyga apicispinata

XML Treatment for Syntozyga spirographa

XML Treatment for Syntozyga similispirographa

## Supplementary materials

Supplementary material 1 Tables S1, S2 Data type table Explanation note Table S1. Species sampled for the molecular analysis. Table S2. Pairwise distances calculated within and between *Syntozyga* species resulting from COI gene dataset. https://binary.pensoft.net/file/528771This dataset is made available under the Open Database License (http://opendatacommons.org/licenses/odbl/1.0/). The Open Database License (ODbL) is a license agreement intended to allow users to freely share, modify, and use this Dataset while maintaining this same freedom for others, provided that the original source and author(s) are credited.Haili Yu
