# Supplementary material for: The genus Syntozyga Lower (Lepidoptera, Tortricidae) in China, with descriptions of two new species
Source: Zookeys. 2021 Apr 6;1028:95–111. doi: 10.3897/zookeys.1028.60297 (PMC8044070; doi:10.3897/zookeys.1028.60297)
Supplement: Supplementary material 1 — Tables S1, S2 [file zookeys-1028-095-s001.docx]

**Notes on *Syntozyga* Lower (Lepidoptera: Tortricidae) from China, with descriptions of two new species**

WENXU YANG, RUIQIN DONG, XUELING SONG & HAILI YU

**Supplementary Data**

**Contents**

Supplementary Table 1: Species sampled for the molecular analysis. 2

Supplementary Table 2: Pairwise distances calculated within and between *Syntozyga* species resulting from COI gene dataset. 3

Supplementary table 1. Species sampled for the molecular analysis.

| **Species** | **Locality** | **Data collected** | **Voucher number** | **GenBank accession number** | **Reference** |
| --- | --- | --- | --- | --- | --- |
| *Syntozyga apicispinata* sp. n. | China, Tibet, Motuo, Bengbeixiang (29°15'N, 95°11'E) | 1.viii.2018 | TORTR008-20 | MW187150 | present study |
| *S. apicispinata* sp. n. | China, Tibet, Motuo, Bengbeixiang (29°15'N, 95°10'E) | 15.viii.2017 | TORTR010-20 | MW187153 | present study |
| *S. apicispinata* sp. n. | China, Tibet,Motuo, Bengbeixiang (29°15'N, 95°11'E) | 12.viii.2017 | TORTR009-20 | MW187152 | present study |
| *S. apicispinata* sp. n. | China, Tibet, Motuo, Bengbeixiang (29°15'N, 95°11'E) | 1.viii.2018 | TORTR011-20 | MW187154 | present study |
| *S. apicispinata* sp. n. | China, Yunnan Pro, Mengla, Bubeng (21°60'N, 101°58'E) | 31.vii.2020 | TORTR001-20 | MW187146 | present study |
| *S. spirographa* | China, Guizhou Pro., Libo, Banzhai village (25°13'N, 108°10'E) | 9.viii.2018 | TORTR004-20 | MW187149 | present study |
| *S. spirographa* | China, Hainan Pro., Dongfang, Lemei village (19°8'N, 108°84'E) | 3.i.2018 | TORTR005-20 | MW187151 | present study |
| *S. spirographa* | China, Guangxi Pro., Yizhou, Pingxin village (24°40'N, 108°21'E) | 16.viii.2011 | TORTR006-20 | MW187155 | present study |
| *S. spirographa* | China, Guizhou Pro., Libo, Banzhai village (25°13'N, 108°10'E) | 24.vii.2019 | TORTR003-20 | MW187148 | present study |
| *S. similispirographa* sp. n. | China, Yunnan Pro., Longling, Xiaoheishan Reserve (24°31'N, 98°50'E) | 30.vii.2015 | TORTR007-20 | MW187156 | present study |
| *S. similispirographa* sp. n. | China, Yunnan Pro., Pu’er, Mt. Yunpan (22°41'N, 100°39'E) | 6.viii.2020 | TORTR002-20 | MW187147 | present study |
| 1. *Psammetalla* | Australia, Queensland, The Millstream Falls 5km SW of Ravenshoe (17°23'S, 145°16'E) | 27.xi.1998 | ANICV1681-11 | KF396180 | Hebert et al. 2013 |
| 1. *Psammetalla* | Australia, Queensland, 2km NE of Weipa (12°38'S, 141°52'E) | 15.xii.1993 | ANICV1683-11 | KF401382 | Hebert et al. 2013 |
| *S. sp* | Australia, Northern Territory, Solar Village, Humpty Doo (12°22'S, 131°3'E) | 28.iii.1998 | ANICV1782-11 | KF399532 | Hebert et al. 2013 |
| *S. sp* | Australia, Northern Territory, Solar Village, Humpty Doo (12°22'S, 131°3'E) | 15.v.1995 | ANICV1781-11 | KF403460 | Hebert et al. 2013 |
| *S. sedifera* | Australia, Northern Territory, 8km NE of Darwin (12°15'S, 130°32'E) | 26.iv.1995 | ANICV1790-11 | KF398928 | Hebert et al. 2013 |
| *S. sedifera* | Australia, Northern Territory, 8km NE of Darwin (12°15'S, 130°32'E) | 26.iv.1995 | ANICV1788-11 | KF405116 | Hebert et al. 2013 |
| *S. sedifera* | Australia, New South Wales, Depot Beach, 16km NE of Batemans Bay (35°37'S, 150°19'E) | 15.iv.1976 | ANICV1789-11 | KF400606 | Hebert et al. 2013 |

Supplementary table 2. Pairwise distance calculated within and between *Syntozyga* species resulting from COI gene dataset.

| **Species** | | **Voucher number** | **Locality** | 1 | 2 | 3 | 4 | 5 | 6 | 7 | 8 | 9 |
| --- | --- | --- | --- | --- | --- | --- | --- | --- | --- | --- | --- | --- |
| 1 | *S. spirographa* | TORTR004-20 | CHN, Guizhou |  |  |  |  |  |  |  |  |  |
| 2 | *S. spirographa* | TORTR003-20 | CHN, Guizhou | 0.000 |  |  |  |  |  |  |  |  |
| 3 | *S. spirographa* | TORTR005-20 | CHN, Hainan | 0.000 | 0.000 |  |  |  |  |  |  |  |
| 4 | *S. spirographa* | TORTR006-20 | CHN, Guangxi | 0.002 | 0.002 | 0.002 |  |  |  |  |  |  |
| 5 | *S. similispirographa* sp. n. | TORTR007-20 | CHN, Yunnan | 0.014 | 0.014 | 0.014 | 0.015 |  |  |  |  |  |
| 6 | *S. smilispirographa* sp. n. | TORTR002-20 | CHN, Yunnan | 0.015 | 0.015 | 0.015 | 0.017 | 0.002 |  |  |  |  |
| 7 | *S. apicispinata*sp. n. | TORTR001-20 | CHN, Yunnan | 0.087 | 0.087 | 0.087 | 0.089 | 0.081 | 0.082 |  |  |  |
| 8 | *S. apicispinata*sp. n. | TORTR008-20 | CHN, Tibet | 0.087 | 0.087 | 0.087 | 0.089 | 0.081 | 0.082 | 0.015 |  |  |
| 9 | *S. apicispinata*sp. n. | TORTR009-20 | CHN, Tibet | 0.087 | 0.087 | 0.087 | 0.089 | 0.081 | 0.082 | 0.015 | 0.000 |  |
| 10 | *S. apicispinata*sp. n. | TORTR010-20 | CHN, Tibet | 0.087 | 0.087 | 0.087 | 0.089 | 0.081 | 0.082 | 0.015 | 0.000 | 0.000 |
| 11 | *S. apicispinata*sp. n. | TORTR011-20 | CHN, Tibet | 0.087 | 0.087 | 0.087 | 0.089 | 0.081 | 0.082 | 0.015 | 0.000 | 0.000 |
| 12 | *S. psammetalla* | ANICV1681-11 | AUS, Queensland | 0.066 | 0.066 | 0.066 | 0.064 | 0.061 | 0.063 | 0.076 | 0.077 | 0.077 |
| 13 | *S. psammetalla* | ANICV1683-11 | AUS, Queensland | 0.063 | 0.063 | 0.063 | 0.061 | 0.058 | 0.060 | 0.076 | 0.074 | 0.074 |
| 14 | *S.* sp. | ANICV1781-11 | AUS, Northern Territory | 0.058 | 0.058 | 0.058 | 0.060 | 0.053 | 0.055 | 0.069 | 0.071 | 0.071 |
| 15 | *S.* sp. | ANICV1782-11 | AUS, Northern Territory | 0.060 | 0.060 | 0.060 | 0.061 | 0.055 | 0.056 | 0.069 | 0.071 | 0.071 |
| 16 | *S. sedifera* | ANICV1790-11 | AUS, Northern Territory | 0.082 | 0.082 | 0.082 | 0.084 | 0.076 | 0.077 | 0.055 | 0.061 | 0.061 |
| 17 | *S. sedifera* | ANICV1789-11 | AUS, Northern Territory | 0.082 | 0.082 | 0.082 | 0.084 | 0.076 | 0.077 | 0.058 | 0.064 | 0.064 |
| 18 | *S. sedifera* | ANICV1788-11 | AUS, New South Wales | 0.081 | 0.081 | 0.081 | 0.082 | 0.075 | 0.076 | 0.053 | 0.060 | 0.060 |

| **Species** | | **Voucher number** | **Locality** | 10 | 11 | 12 | 13 | 14 | 15 | 16 | 17 | 18 |
| --- | --- | --- | --- | --- | --- | --- | --- | --- | --- | --- | --- | --- |
| 11 | *S. apicispinata*sp. n. | TORTR011-20 | CHN, Tibet | 0.000 |  |  |  |  |  |  |  |  |
| 12 | *S. psammetalla* | ANICV1681-11 | AUS, Queensland | 0.077 | 0.077 |  |  |  |  |  |  |  |
| 13 | *S. psammetalla* | ANICV1683-11 | AUS, Queensland | 0.074 | 0.074 | 0.003 |  |  |  |  |  |  |
| 14 | *S.* sp. | ANICV1781-11 | AUS, Northern Territory | 0.071 | 0.071 | 0.023 | 0.023 |  |  |  |  |  |
| 15 | *S.* sp. | ANICV1782-11 | AUS, Northern Territory | 0.071 | 0.071 | 0.023 | 0.023 | 0.003 |  |  |  |  |
| 16 | *S. sedifera* | ANICV1790-11 | AUS, Northern Territory | 0.061 | 0.061 | 0.074 | 0.071 | 0.071 | 0.073 |  |  |  |
| 17 | *S. sedifera* | ANICV1789-11 | AUS, Northern Territory | 0.064 | 0.064 | 0.077 | 0.074 | 0.071 | 0.073 | 0.014 |  |  |
| 18 | *S. sedifera* | ANICV1788-11 | AUS, New South Wales | 0.060 | 0.060 | 0.076 | 0.073 | 0.073 | 0.074 | 0.002 | 0.015 |  |

Supplementary table 2. (Continued).
